# Lateral Tarsoplasty Combined with a Full-Thickness Skin Graft for Managing Severe Lower Eyelid Ectropion Following the Subciliary Approach for Infra-Orbital Rim Fracture: A Retrospective Observational Study

**DOI:** 10.3390/life14030314

**Published:** 2024-02-28

**Authors:** Wonseok Cho, Chang Gyun Kim, Eun A Jang, Kyu Nam Kim

**Affiliations:** 1 Department of Plastic and Reconstructive Surgery, Kangbuk Samsung Hospital, Sungkyunkwan University School of Medicine, Seoul 03181, Republic of Korea; wscws26@naver.com (W.C.); insane.blood.a@gmail.com (E.A.J.); 2 Face Plastic Surgery Clinic, Daejeon 35230, Republic of Korea; pskyudong87@naver.com

**Keywords:** ectropion, orbital fracture, zygoma fractures, eyelids, skin transplantation

## Abstract

Subciliary incision is a common approach for facial fracture surgery; however, it has a higher incidence of lower lid ectropion, which can be particularly challenging for beginning surgeons to manage. This study reports the usage of lateral tarsoplasty combined with a full-thickness skin graft (FTSG) to correct severe ectropion following the subciliary approach for infra-orbital rim fractures. We retrospectively reviewed all facial fracture cases involving infra-orbital rim fractures through a subciliary approach treated in our department between March 2021 and May 2023. Electronic medical records and clinical digital photographs of patients who met the inclusion criteria were reviewed. After reviewing 196 cases that used the subciliary approach, we found 6 patients (3.06%; 4 males and 2 females; mean age, 68.5 ± 4.89 years) with postoperative severe ectropion managed using lateral tarsoplasty and FTSG. The mean ectropion development and correction times after facial fracture surgery were 0.78 ± 0.24 and 0.91 ± 0.37 months, respectively. At the 12-month follow-up, all patients showed favorable outcomes, and the position of their lower eyelids was well maintained without ectropion recurrence. Based on these successful outcomes, lateral tarsoplasty combined with FTSG is proposed to be an effective and straightforward method for managing lower eyelid ectropion caused by facial fracture surgery.

## 1. Introduction

Subciliary incision is one of the most frequently used approaches for facial bone fractures, particularly orbital floor and zygomaticomaxillary complex fractures involving the inferior orbital rim [1]. This approach allows a broad view of the operative field, facilitating precise fracture reduction [1]. However, it is linked to postoperative eyelid complications, including lower eyelid malposition, sclera show, lid edema, entropion, and ectropion [1,2,3,4,5,6]. Ectropion, which is characterized by an outward turning of the eyelid margin, exposes the inner eyelid surface, increasing its susceptibility to irritation. Previous studies have reported the incidence of ectropion or sclera show with the subciliary approach ranging from 6% to 20% [1,2,3,4,7]. Mild to moderate ectropion may ameliorate with time and gentle massage; however, severe cases usually necessitate surgical intervention [1,7,8,9,10]. Notably, several corrective methods have been documented, including lateral musculopexy, canthopexy, canthoplasty, the tarsal strip technique, and tarsoplasty [1,7,8,9,10].

Nonetheless, a universally accepted technique for ectropion correction remains elusive. Therefore, this retrospective study aimed to describe our experiences with lateral tarsoplasty combined with a full-thickness skin graft (FTSG) in addressing severe ectropion after the subciliary approach in managing infra-orbital rim fractures. Generally, severe ectropion is defined as a condition in which the entire length of the eyelid is turned out. In this study, we performed that surgical correction of severe ectropion that either did not respond to non-surgical management or resulted in persistent discomfort in the patient’s life, despite a partial response to non-surgical management. Lateral tarsoplasty was first developed by Chung-Sheng Lai and was used to correct lower eyelid ectropion [7]; furthermore, our senior author (the corresponding senior author of this study) first applied this technique, combined with FTSG, to address severe ectropion complicated by the subciliary approach used in managing infra-orbital rim fracture. To our knowledge, our study may be the first case series regarding the correction of severe ectropion following the subciliary approach for the management of facial bone fractures in Korea. Therefore, we postulate that this technique is valuable in managing such challenging scenarios.

## 2. Materials and Methods

### 2.1. Ethical Compliance

The Institutional Review Board of Kangbuk Samsung Hospital (approval number: 2023-12-011; date of approval: 13 December 2023) approved this study. All research procedures followed the ethical guidelines outlined in the 1975 Declaration of Helsinki, and written informed consent was obtained from all patients.

### 2.2. Patient Selection

A retrospective review was conducted for all traumatic facial fracture cases involving infra-orbital rim fractures that were approached through subciliary incision in our department between March 2021 and May 2023. We enrolled patients who underwent lateral tarsoplasty combined with FTSG to address severe ectropion after the subciliary approach for managing infra-orbital rim fractures. Furthermore, we excluded patients who received only non-surgical managements, such as triamcinolone acetonide intralesional injection, for managing ectropion developed after the subciliary approach. Patients who developed ectropion that was spontaneously alleviated over time were also excluded. In this study, surgical correction of ectropion using lateral tarsoplasty combined with FTSG was performed on patients with severe ectropion unresponsive to non-surgical managements, such as gentle massage and triamcinolone acetonide intralesional injection, or those who opted for surgical correction due to persistent discomfort despite the condition’s partial response to non-surgical management. Retrospective data analysis of the enrolled patients was performed using the patient’s electronic medical records and clinical digital photographs. The data comprised facial bone fracture type, ectropion development time after open reduction and internal fixation (ORIF), ectropion correction time after ORIF, postoperative (ectropion correction) complications, and final follow-up periods.

### 2.3. Surgical Techniques (Lateral Tarsoplasty Combined with Full-Thickness Skin Graft)

**Figure 1:**
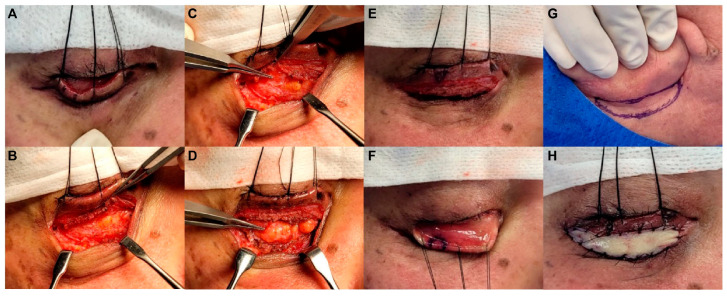
Ectropion correction surgery procedures, including lateral tarsoplasty combined with a full-thickness skin graft (FTSG). (**A**) The incision was made through the previous subciliary incision line used for the previous facial fracture surgery. (**B**) Dissection proceeded above the orbital septum. (**C**,**D**) The surrounding scar tissues were removed, and the adhesion was released. (**E**,**F**) Lateral tarsoplasty, including a full-thickness pentagonal skin-tarsal-conjunctival resection with 5–7 mm width, was performed at approximately 5 mm medial to the lateral canthus. (**G,H**) FTSG from the postauricular area was performed between the skin incision areas.

**Figure 2:**
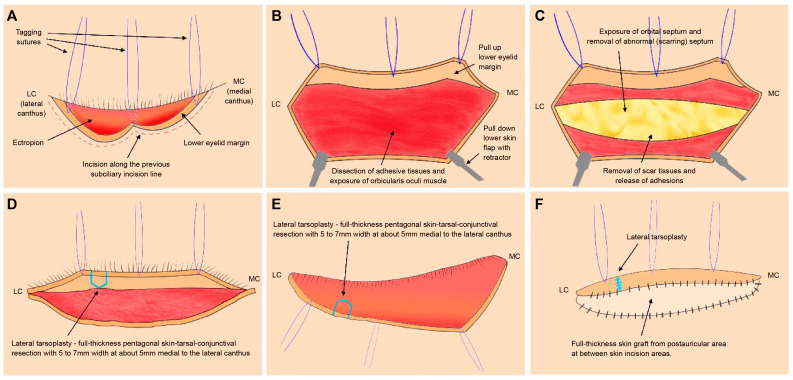
A schematic illustration of the lateral tarsoplasty combined with a full-thickness skin graft (FTSG) for correcting severe lower eyelid ectropion following the subciliary approach for infra-orbital rim fracture. (**A**) An incision was made through the previous subciliary incision line used for the previous facial fracture surgery. (**B**,**C**) Dissection was performed above the orbital septum, the surrounding scar tissues were removed, and the adhesion was released. (**D**,**E**) Lateral tarsoplasty, including a full-thickness pentagonal skin-tarsal-conjunctival resection with 5–7 mm width, was performed at approximately 5 mm medial to the lateral canthus. (**F**) Following the lateral tarsoplasty, FTSG from the postauricular area was performed between the skin incision areas.

In this study, lateral tarsoplasty combined with FTSG was conducted in all cases by a single surgeon (our senior surgeon, who is this study’s corresponding author). Notably, all surgical procedures were conducted under local anesthesia. Following infiltration with 1% lidocaine hydrochloride and 1:100,000 epinephrine solution, a skin incision was made along the subciliary incision line from the previous ORIF (Figure 1A). Dissection proceeded from the orbicularis oculi muscle to the orbital septum (Figure 1B). Next, the surrounding scar tissues were excised, and scar contractures and adhesion tissues were released (Figure 1C,D). Lateral tarsoplasty was performed approximately 5 mm medial to the lateral canthus using a full-thickness pentagonal skin–tarsal–conjunctival resection with a width of 5–7 mm (Figure 1E,F). At this stage, we applied the suturing method used in a previous study as follows [7]. First, for reapproximating both resected ends of the tarsus, we applied two 6-0 Vicryl simple interrupted sutures, while confirming an accurate realignment of the gray line [7]. Next, the tarsal repair, using a 6-0 Vicryl suture, was started by inserting the needle from the tarsus lower end bevel to its most superior medial border using the outside-in technique, then re-entering the needle at the same level of the opposite tarsus using the inside-out technique, which could make the tarsus push-up when tying the suture [7]. After reapproximating the orbicularis oculi muscle with a 6-0 Vicryl suture, skin closure using a 6-0 Nylon suture was finally performed, with an emphasis on achieveing the correct realignment of the gray line [7]. An FTSG from the postauricular area was placed between the skin incision areas (Figure 1G,H), and a tie-over dressing was applied. Figure 2 shows a schematic representation illustrating the lateral tarsoplasty combined with FTSG performed for severe ectropion correction in our cases.

### 2.4. Evaluation of the Postoperative Cosmetic Outcomes Using the Patient and Observer Scar Assessment Scale (POSAS)

The Patient and Observer Scar Assessment Scale (POSAS) was used to assess the final cosmetic outcomes [11,12]. At the last follow-up held 12 months after the ectropion correction surgery, a single observer (our senior author) evaluated the Observer Scar Assessment Scale (OSAS), comprising six items scored on a 10-point scale, as follows: vascularity, pigmentation, thickness, pliability, relief, and surface area [11,12]. Each patient self-assessed the final scar using the Patient Scar Assessment Scale (PSAS), which estimates six scar characteristics scored on a 10-point scale, as follows: pain, itching, color, stiffness, thickness, and irregularity [11,12]. The total scores ranged from 6 to 60 in the OSAS and PSAS, with a span of 6 and 60, indicating perfectly normal skin ranging to the worst imaginable scar, respectively [11,12]. After scoring the items, the observer and the patient rated the overall scar appearance to obtain an objective scar rating and overall patient satisfaction based on a 10-point visual analog scale, ranging from poor to excellent [11,12].

## 3. Results

We identified 196 cases that used the subciliary approach for the ORIF of facial bone fracture involving the infra-orbital rim fracture; postoperative ectropion conservatively managed, without surgical correction, was found in 24 (12.24%) cases, and postoperative severe ectropion, managed using lateral tarsoplasty combined with FTSG, was found in 6 (3.06%) cases. In total, six (four males and two females) patients, aged 62–75 years (mean age, 68.5 ± 4.89 years), were included in this study. Among the six severe ectropion cases, the subciliary approach was used for orbito-zygomaticomaxillary complex and blow-out fractures in four and two patients, respectively. Notably, none of the cases involved laceration wounds at or around the subciliary incision site. The mean ectropion development and correction time after ORIF was 0.78 ± 0.24 (range, 0.5–1) and 0.91 ± 0.37 (range, 1.5–2.5) months, respectively. Notably, no patients experienced postoperative (after ectropion correction surgery) complications, such as wound infection, dehiscence, hematoma, or recurrent ectropion. The final follow-up period in this study was 12 months after ectropion correction surgery in all enrolled patients. At their final follow-up, all patients were fairly satisfied with the outcomes, and the position of their lower eyelids was well maintained, without the recurrence of ectropion. Table 1 summarizes the clinical data of patients in this study. Table 2 and Table 3 show the PSAS and OSAS assessment results, respectively. The mean PSAS and OSAS total scores were 13.16 ± 1.60 and 17.66 ± 0.51, respectively. Additionally, the mean overall patient satisfaction and objective scar ratings were 4.16 ± 0.75 and 4.66 ± 0.51, respectively. The subsequent section (case presentations) describes the representative cases illustrating the results of lateral tarsoplasty combined with the FTSG technique for correcting severe ectropion after the subciliary approach in managing infra-orbital rim fractures.

### 3.1. Case Presentations

#### 3.1.1. Case 1

**Figure 3:**
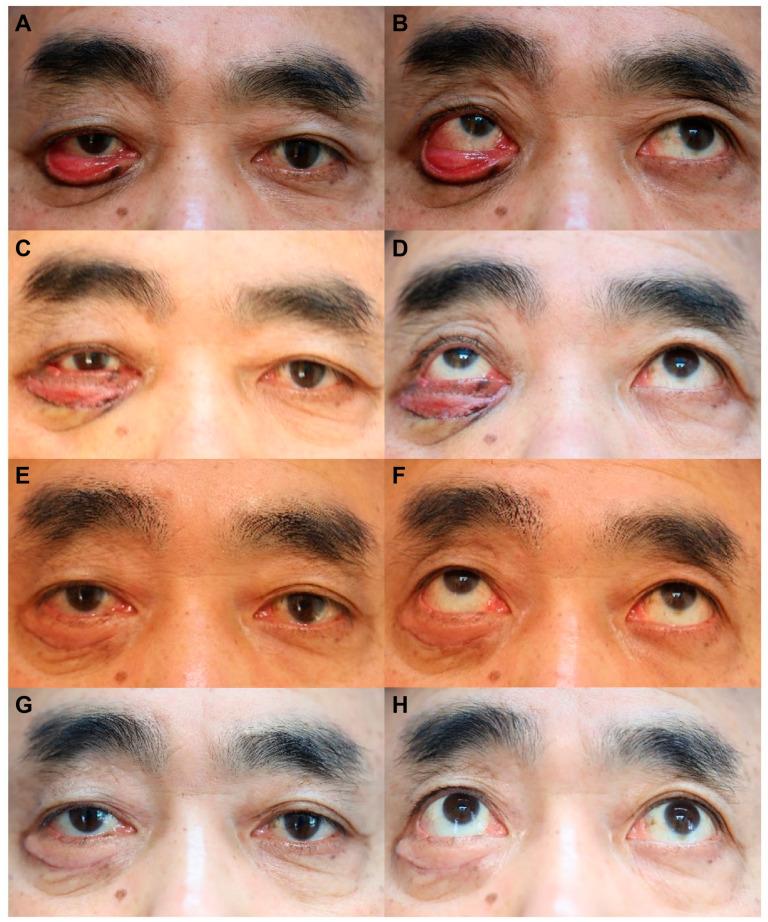
Serial follow-up photographs of case 1, a 62-year-old man who showed severe lower eyelid ectropion after open reduction and internal fixation (ORIF) through a subciliary approach for infra-orbital rim fracture, before and after the ectropion correction surgery. (**A**,**B**) Preoperative (ectropion correction surgery) photographs at the 2-month follow-up after ORIF. (**C**,**D**) Postoperative (ectropion correction surgery) photographs at the 10-day follow-up. (**E**,**F**) Postoperative (ectropion correction) photographs at the 6-month follow-up. (**G**,**H**) Postoperative (ectropion correction) photographs at the 12-month follow-up.

A 62-year-old male patient presented with ectropion of the lower eyelid at a 20-day follow-up after an ORIF performed through the subciliary approach for managing infra-orbital rim fracture. The ectropion worsened over time, despite an initial encouragement for gentle massage. Lateral tarsoplasty combined with FTSG was performed at the 1.5-month follow-up after ORIF (Figure 3A,B). No postoperative complications were observed, with successful skin graft integration at the 5-day follow-up. Subsequent evaluations at 10 days (Figure 3C,D), 6 months (Figure 3E,F), and 12 months (Figure 3G,H) revealed no ectropion relapse, and the patient was satisfied with the outcome. At the final follow-up, the PSAS total, OSAS total, overall patient satisfaction, and objective scar rating scores were 11, 17, 3, and 4, respectively.

#### 3.1.2. Case 2

**Figure 4:**
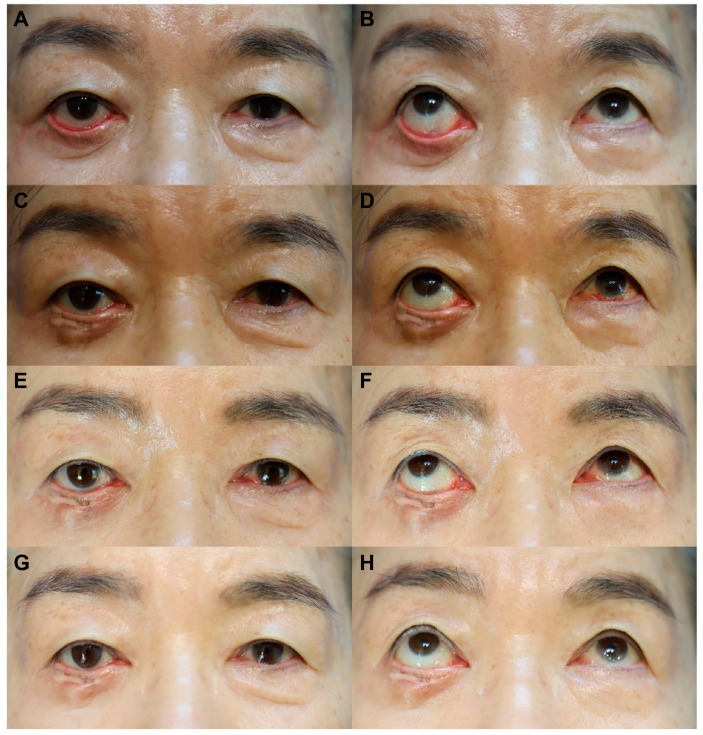
Serial follow-up photographs of case 2, a 75-year-old female patient who showed severe lower eyelid ectropion after open reduction and internal fixation (ORIF) using the subciliary approach for infra-orbital rim fracture, before and after the ectropion correction surgery. (**A**,**B**) Preoperative (ectropion correction surgery) photographs at the 2.5-month follow-up after ORIF. (**C**,**D**) Postoperative (ectropion correction surgery) photographs at the 2-month follow-up. (**E**,**F**) Postoperative (ectropion correction) photographs at the 5-month follow-up. (**G**,**H**) Postoperative (ectropion correction) photographs at the 12-month follow-up.

A 75-year-old woman showed ectropion of the lower eyelid at the 1-month follow-up after an ORIF through the subciliary approach for infra-orbital rim fracture. Serial intralesional injection of triamcinolone acetonide was promptly initiated for 1 month, improving the ectropion degree. However, the patient opted for surgical correction due to persistent discomfort. Subsequently, lateral tarsoplasty combined with FTSG was performed at the 2.5-month follow-up after ORIF (Figure 4A,B). There were no postoperative complications, and successful skin graft integration was observed at the 5-day follow-up. Subsequent assessments at 2 (Figure 4C,D), 5 (Figure 4E,F), and 12 months (Figure 4G,H) demonstrated no ectropion relapse, and the patient was satisfied with the outcomes. At the final follow-up, the PSAS total, OSAS, overall patient satisfaction, and objective scar rating scores were 13, 8, 4, and 4, respectively.

## 4. Discussion

This study outlines the successful correction of severe ectropion complicating the subciliary approach for infra-orbital rim fractures using lateral tarsoplasty combined with FTSG conducted by a single surgeon (this study’s corresponding author) in six consecutive patients, yielding favorable outcomes. The subciliary approach is a conventional and effective method for reducing infra-orbital rim and zygomaticomaxillary complex fractures [1,2,5,6]. Particularly, in the case of facial fractures involving the infra-orbital rim that should be sufficiently exposed, the subciliary approach is commonly used because it can allow wider exposure than the transconjunctival approach and may achieve less noticeable postoperative scarring than the subtarsal or infra-orbital approach [2,5,6]. However, the frequent development of lower eyelid malposition, particularly severe ectropion, is a prevalent long-term complication associated with this approach. These occurrences usually resist conservative interventions, such as a gentle massage and a triamcinolone acetonide injection [1,2,3,4,5,6]. The lower eyelid is a crucial structure of the face, both cosmetically and functionally. It not only provides functions associated with eyeball protection, facilitating eye opening, and maintaining eye surface moisture, but it also plays an important cosmetic role in determining a person’s expressions and features, due to its central location in the face. Therefore, complications of the lower eyelid, such as ectropion, can be challenging for patients and surgeons, and such complications should be approached cautiously and resolved by considering both functional and cosmetic aspects. In our study, the incidence of postoperative ectropion following the subciliary approach for managing facial fracture surgery was 12.24%, consistent with the outcomes of previous studies [1,2,3,4,7]. However, some studies have reported on the incidence of ectropion requiring surgical interventions after the use of the subciliary approach [3,13,14,15,16,17,18,19,20]. A meta-analysis study, with 470 patients who underwent the subciliary approach for facial fracture surgery, revealed 68 (14%), 54 (11%), and 14 (3%) patients, respectively, who developed ectropion, whose ectropion was conservatively managed, and whose ectropion was operatively managed [3]. In our study, 6 of the 196 patients (3.06%) underwent surgical correction of ectropion through lateral tarsoplasty combined with FTSG, which was consistent with the previous study’s results.

Ectropion following the subciliary approach results from a complex interplay of factors, including scar retraction linked to inadequate skin and muscle manipulation, lower eyelid laxity associated with the lateral canthal tendon’s inherent laxity or disinsertion, and subsequent scarring and adhesion of middle lamellar structures, such as the orbital septum and fat [1,3,4,7]. Notably, the subciliary approach for facial bone fracture surgery differs from aesthetic blepharoplasty, where ancillary procedures, such as lateral canthopexy, lateral canthoplasty, and midface lift, are commonly used to reduce the incidence of postoperative ectropion. These additional procedures are not routinely performed in facial bone fracture surgery. Moreover, substantial subperiosteal dissection following the subciliary approach increases the vertical vector of gravity on the anterior lamella structures, making the lower eyelid more vulnerable to the occurrence of ectropion.

Notably, various surgical options exist for correcting ectropion, and their selection should be tailored to the underlying etiology [7]. Procedures addressing lower eyelid laxity include lateral musculopexy, canthopexy, canthoplasty, the tarsal strip technique, and wedge resection of the lower eyelid [7,8,9,10,21,22,23,24]. Ectropion due to scar formation in the lower eyelid lamella structures can be managed by removing scar tissue, releasing scar contractures, and subsequent skin replacement [7,25,26,27].

In our cases, we opted for lateral tarsoplasty combined with FTSG, distinguishing it from lateral canthoplasty by avoiding the lateral canthal ligament [7]. This approach mitigates the risk of misplacing the canthal tendon anchoring points, preventing the unintended displacement of the eyelid and leading to the malposition or lateral ectropion of the lower eyelid [7]. Notably, the subciliary approach for managing infra-orbital rim fractures preserves the canthal ligament structures, avoids tissue deficits, and ensures proper anatomical repositioning through meticulous surgical techniques [1]. Therefore, we believe that lateral tarsoplasty suffices as a corrective modality for ectropion following the subciliary approach for managing infra-orbital rim fractures because of the preservation of the canthal ligament structures through scrupulous and elaborate surgical procedures. Lateral tarsoplasty provides horizontal lid tightening, without manipulating the lateral canthal structures, through a full-thickness pentagonal skin-tarsal-conjunctival resection [7]. The resection location, 5 mm medial to the lateral canthus, avoids the deformation of the lateral canthal structures [7]. As originally mentioned by Chung-Sheng Lai, the overlapping test, which is performed by gently pulling the lateral tarsus medially and the medial tarsus laterally using two forceps immediately after the first incision, can provide an accurate measurement of the amount of tarsus to be resected [7]. The width of the resected tarsus in our study, which was tailored to the severity of ectropion, ranged from 5 to 7 mm, compared with the 3–10 mm width of the resected tarsus in the previous study conducted by Chung-Sheng Lai [7]. FTSG, performed immediately after lateral tarsoplasty, is crucial in ectropion correction, preventing subsequent cicatricial ectropion and providing substantial structural support by securing the vertical length of the lower eyelid skin as a spacer graft. Successful correction of severe ectropion following a subciliary approach for managing infra-orbital rim fractures, without recurrence in all cases, could be achieved using a combination of these procedures, including lateral tarsoplasty, with its horizontal lid-tightening effect, and FTSG, with its spacer graft role.

Lateral tarsoplasty is not a new technique for ectropion correction in the lower eyelid. A previous study provided a detailed description of this technique in managing ectropion and laxity of the lower eyelid [7]. As previously noted, the abovementioned study also explained the benefits of lateral tarsoplasty, which include its effectiveness and efficiency, without compromising the integrity of the lateral canthal complex, both of which can be attributed to its accuracy and ease in pre- and intra-operative design, its short procedural time, and its long-lasting effect [7]. The study mentioned above also deduced that lateral tarsoplasty is a simple and effective method that can be performed to correct, and even prevent, ectropion of the lower eyelids while preserving the lateral canthal ligament integrity, thereby preventing lower eyelid malposition in the case of moderate and severe laxity of the lower eyelids [7]. The characteristics of our research which distinguish it from the previously mentioned study are as follows. The previous study used the lateral tarsoplasty technique for ectropion correction in cases of lower blepharoplasty or as a preventive treatment for lower eyelid laxity in cases of senile change of the lower eyelids [7]. In contrast, we applied the lateral tarsoplasty technique for the correction of more severe ectropion following the subciliary approach for managing facial bone fractures. Furthermore, as previously mentioned, we combined the lateral tarsoplasty technique with FTSG to prevent subsequent cicatricial ectropion and to provide substantial structural support, which resulted in no recurrence of ectropion in our cases. To evaluate the results of the follow-up observations, including the level of patient satisfaction with the postsurgical outcomes, they used the 5-point Likert scale, which is a relatively simple and intuitive evaluation tool [7]. In our study, we used the POSAS, which is a widely used scar assessment scales devised by Draaijers et al. in 2004, that can provide a more suitable, reliable, and comprehensive scar evaluation for all types of scars [11]. Since the impression of the scar’s appearance is determined by subjective opinions, it is appropriate to evaluate the scar through the subjective scale of the patient (PSAS) and that of a third party (OSAS). Thus, our study can more systematically evaluate the results of ectropion correction surgery than the previous study, from the perspective of both patients and surgeons.

We achieved successful severe ectropion correction in six cases using lateral tarsoplasty combined with FTSG; however, this study had some limitations. Conservative measures, such as gentle massage and triamcinolone acetonide injection, integral to ectropion correction [1], were not thoroughly detailed. A previous study mentioned that ectropion due to postoperative scarring and the retraction of the lower eyelid is most pronounced 1–3 months postoperatively, and that the symptoms gradually reduced over 6 months [28,29]. Additionally, the abovementioned study observed that the follow-up regarding ectropion should be at least 6 months [28]. However, because of the discomfort in daily life due to the accompanying symptoms, such as excessive tearing, dry eyes, inability to close the eyelids completely, and eye pain, it can be difficult for patients to wait 6 months for ectropion to improve spontaneously, without surgical intervention. Therefore, the timing for the surgical correction of ectropion may be determined based on the severity of the ectropion-related symptoms and the patient’s need. However, future studies should explore these conservative interventions, including their application sequence and duration before surgical correction.

Furthermore, our study included only Asians (Korean patients), and physical factors, such as scar tissue weight, gravity effect on the surrounding tissues, and tension force effect on skin closure; or skin properties, including thickness, roughness, and hydration, could not be analyzed [30,31]. Additionally, our study, including only six cases, with a relatively small sample size and a non-randomized retrospective design, introduces potential selection bias and confounding factors [32,33]. Moreover, our study is not a comparative study but a descriptive study, with one surgeon’s (this study’s corresponding author) case series. Therefore, prospective large-scale studies with diverse comparison groups are required to validate our findings [32,33].

Finally, just as previous studies have demonstrated the effectiveness of using lateral tarsoplasty as a preventive treatment modality for ectropion in aesthetic blepharoplasty [7], it will be necessary to investigate the effectiveness of lateral tarsoplasty in preventing the development of ectropion following the subciliary approach to access facial fractures. In other words, although it was not performed in the present study, we plan to conduct preoperative tests for evaluating the severity of lower lid laxity, such as a distraction test and a snap-back test [7], and simultaneously perform lateral tarsoplasty with facial fracture surgery using the subciliary approach in patients with high risk of developing postoperative ectropion. Thus, we will verify the preventive effectiveness of lateral tarsoplasty in the subciliary approach for facial fracture surgery.

## 5. Conclusions

Considering the study’s limitations, this investigation is significant as the inaugural consecutive case series by a single surgeon documenting the successful correction of severe ectropion following the subciliary approach for managing infra-orbital rim fractures using lateral tarsoplasty combined with FTSG in Korea. Our findings propose lateral tarsoplasty combined with FTSG as a viable reconstructive option for correcting severe lower eyelid ectropion. This recommendation may be based on the procedure’s technical simplicity, safety, and its favorable outcomes in selected cases. However, future research endeavors, including prospective studies using larger cohorts and diverse patient groups, should further validate this corrective approach’s efficacy and its broader applicability.

## Figures and Tables

**Table 1:** Summary of patient data.

Case Number	Sex/Age	Type of Facial Bone Fracture	Ectropion Development Time after ORIF (Months)	Ectropion Correction Time after ORIF (Months)	Postoperative (Ectropion Correction Surgery) Complications	Final Follow-Up Periods (Months)
1	M/62	Orbito-ZMC Fx	0.6	1.5	None	12
2	F/75	Orbito-ZMC Fx	1	2.5	None	12
3	M/68	Blow-out Fx	0.5	2	None	12
4	M/70	Orbito-ZMC Fx	1	2	None	12
5	F/64	Blow-out Fx	0.6	1.5	None	12
6	M/72	Orbito-ZMC Fx	1	2	None	12

M, male; F, female; Orbito-ZMC Fx, orbito-zygomaticomaxillary complex fracture; Fx, fracture.

**Table 2:** Patients’ Patient Scar Assessment Scale score data.

Case Number	Pain	Itching	Color	Stiffness	Thickness	Irregularity	PSAS Total Score	Overall Patient Satisfaction Rating
1	1	1	3	2	2	2	11	3
2	2	1	2	2	3	3	13	4
3	1	1	3	2	3	3	13	4
4	2	1	3	2	2	2	12	4
5	2	1	3	3	3	3	15	5
6	2	1	3	3	3	3	15	5

PSAS, Patient Scar Assessment Scale.

**Table 3:** Patients’ Observer Scar Assessment Scale (OSAS) score data.

Case Number	Vascularity	Pigmentation	Thickness	Pliability	Relief	Surface Area	OSAS Total Score	Objective Scar Rating
1	2	4	3	3	2	3	17	4
2	3	3	3	3	3	3	18	4
3	2	3	3	3	3	4	18	5
4	3	4	3	3	2	3	18	5
5	2	3	3	3	3	3	17	5
6	3	3	3	3	3	3	18	5

OSAS, Observer Scar Assessment Scale.

## Data Availability

The data presented in this study are available upon request from the corresponding author. The data are not publicly available due to privacy restrictions.
